# An effect comparison of alendronate and teriparatide in patients with glucocorticoid-induced osteoporosis: A protocol for systematic review and meta-analysis

**DOI:** 10.1097/MD.0000000000032090

**Published:** 2022-12-02

**Authors:** Na Liang, Shuang Zhang, Shuang Wang, Juan Ma

**Affiliations:** a Department of Orthopaedics, Shengjing Hospital of China Medical University, Liaoning, China.

**Keywords:** alendronate, glucocorticoid, meta-analysis, osteoporosis, systematic review, teriparatide

## Abstract

Glucocorticoid-induced osteoporosis is the most common secondary cause of osteoporosis and the resulting fractures cause significant morbidity. Oral bisphosphonates are currently regarded as first line options on the grounds of their low cost. However, teriparatide has been shown to be superior in its effects on bone mineral density and vertebral fracture risk in glucocorticoid-treated individuals with osteoporosis. We conducted a protocol for systematic review and meta-analysis to assess the effectiveness of alendronate and teriparatide in patients with glucocorticoid-induced osteoporosis.

**Methods:**

The study protocol has been registered on international prospective register of systematic review (PROSPERO registration number: CRD42022371561). The procedure of this protocol will be conducted according to the Preferred Reporting Item for Systematic Review and Meta-analysis Protocols guidance. PubMed, EMBASE, MEDLINE, the Cochrane Library, Chinese National Knowledge Infrastructure, Chinese Biomedical Literature Database, Wanfang Database, ClinicalTrials.gov trials registry, and Chinese Clinical Trial Registry will be searched from January 1980 to November 2022. Two authors will assess methodological quality of included studies separately by the Cochrane collaboration’s risk of bias tool. We will apply RevMan 5.4 software for statistical analysis.

**Results:**

This study will provide a high-quality comprehensive evaluation of the efficacy and safety of alendronate and teriparatide for treating patients with glucocorticoid-induced osteoporosis.

**Conclusion:**

The conclusion of our systematic review will provide evidence to judge whether teriparatide is an effective intervention for patients with glucocorticoid-induced osteoporosis.

## 1. Introduction

Glucocorticoid-induced osteoporosis is the most common secondary cause of osteoporosis.^[1,2]^ It has been estimated that up to 1% to 2% of the general population is receiving long term oral glucocorticoids therapy.^[3]^ Following initiation of oral glucocorticoids, rapid bone loss occurs, and fracture risk increases within a few months in a dose-dependent manner. Information deriving from population-based epidemiologic studies indicates that up to 30% to 40% of individuals using long-term glucocorticoids may experience a fragility fracture.^[4,5]^ Since the global population is aging, osteoporosis and fragility fracture have become public health concerns because of its considerable morbidity, excess mortality, and great risk of disability.^[6]^

These adverse effects are due to inhibition of bone formation accompanied by an early but transient increase in bone resorption. Multiple mechanisms underlie these changes in bone remodeling; direct effects include upregulation of PPARγR2, increased expression of sclerostin and increased RANKL/OPG ratio, whilst hypogonadism, altered renal and intestinal calcium handling, and reduced production of insulin-like growth factor 1 also contribute.^[7–9]^

Fracture risk assessment should be performed as soon as possible after glucocorticoids are initiated and bone protective therapy started promptly in individuals at high-risk, with calcium and vitamin D supplements where appropriate.^[10,11]^ Oral bisphosphonates are currently regarded as first line options on the grounds of their low cost. Teriparatide had dual, time-dependent effects on bone resorption and bone formation, and intermittent teriparatide could directly increase osteoblasts activity and indirectly increase bone resorption.^[12]^ It has been shown to be superior in its effects on bone mineral density (BMD) and vertebral fracture risk in glucocorticoid-treated individuals with osteoporosis and should be considered as an alternative first line option in high-risk patients.^[13]^ At present, there is no reliable evidence regarding the comparisons of anti-osteoporosis effects between alendronate and teriparatide. Thus, we conducted a protocol for systematic review and meta-analysis to assess the effectiveness of alendronate and teriparatide in patients with glucocorticoid-induced osteoporosis.

## 2. Methods

### 2.1. Registration

The study protocol has been registered on international prospective register of systematic review (PROSPERO registration number: CRD42022371561). The procedure of this protocol will be conducted according to the Preferred Reporting Item for Systematic Review and Meta-analysis Protocols guidance.^[14]^ This systematic review will not require ethical approval because there are no data used in our study that are linked to individual patient data.

### 2.2. Inclusion and exclusion criteria

#### 2.2.1. Type of study

Randomized controlled trials comparing alendronate and teriparatide in patients with glucocorticoid-induced osteoporosis will be included. Studies will be excluded if they are: Nonrandomized controlled trials, literature review, case-control trials, and animal research literature; Studies with repeated publication, unclear outcome measures, and obvious data errors; Studies with fewer than 10 samples.

#### 2.2.2. Type of participant

Patients aged 45 to 85 years old were eligible for enrollment if they had a BMD T-score of −2.5 or less at the lumbar spine, total hip, or femoral neck or a BMD T-score of −1.0 or less at either of the above sites with a history of one or more fragility fractures at the vertebra, hip, proximal humerus, or distal radius.

#### 2.2.3. Type of intervention and control

Intervention group received oral alendronate and control group received subcutaneous injection of teriparatide. There will be no restrictions with respect to dosage, frequency, duration, or follow-up time of treatment.

#### 2.2.4. Study outcomes

The primary endpoint of the study was confirmation of non-inferiority of teriparatide to alendronate for the mean percentage change in BMD at the lumbar spine from baseline to 48 weeks. Secondary efficacy end point was superiority test comparing teriparatide and alendronate for the mean percentage change in BMD at the lumbar spine from baseline to 48 weeks, which was evaluated only if noninferiority was demonstrated. Additional end points included the mean percentage changes in BMD at the lumbar spine from baseline to 24 weeks; the mean percentage changes in BMD at total hip from baseline to 24 and 48 week.

### 2.3. Search strategy

PubMed, EMBASE, MEDLINE, the Cochrane Library, Chinese National Knowledge Infrastructure, Chinese Biomedical Literature Database, Wanfang Database, ClinicalTrials.gov trials registry, and Chinese Clinical Trial Registry will be searched from January 1980 to November 2022. A combination of subject words and free text words will be applied in the searches. The language is limited to Chinese and English. The search terms are shown in Table 1.

**Table 1:** Search strategy of PubMed.

#1 "random*"[Text Word] OR allocation[Text Word] OR "random allocation"[Text Word] OR placebo[Text Word] OR single blind[Text Word] OR double blind[Text Word] OR "randomized controlled trial*"[Text Word] OR RCT[Text Word]
#2 randomized controlled trial[Publication Type]
#3 #1 OR #2
#4 animals NOT humans
#5 #3 NOT #4
#6 "osteoporosis" [Text Word] OR "bone rarefaction"[Text Word] OR "rarefaction of bone" [Text Word]
#7 "glucocorticoids" [Text Word] OR "dexamethasone"[Text Word] OR "methylprednisolone" [Text Word] OR "betamethasone" [Text Word]
#8 "alendronate" [Text Word] OR "bisphosphonates"[Text Word]
#9 "teriparatide" [Text Word] OR "teriparatideacetate"[Text Word]
#10 #5 AND #6 AND #7 AND #8 AND #9

### 2.4. Study selection

**Figure 1.**
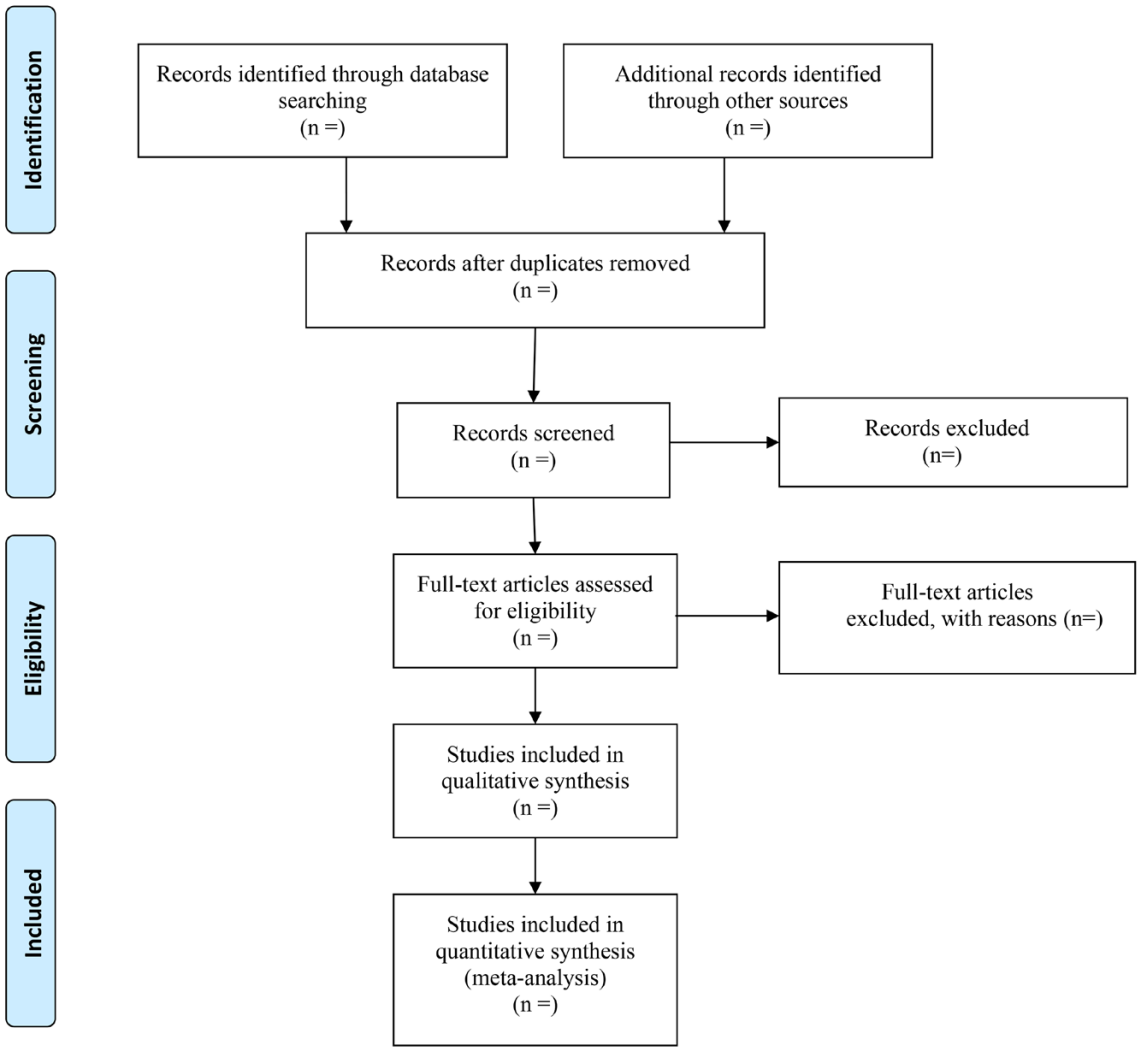
Flow diagram of study selection.

The literature will be retrieved according to the retrieval strategy, then imported them into the literature management software. The research on duplicate titles was deleted, and obviously irrelevant literature was excluded by reading titles and abstracts. The above steps were performed independently by 2 researchers. Any disagreements will be resolved by discussion with third researchers. The researchers will record all studies that do not meet the inclusion criteria and provide the rationale for their exclusion. Details of the selection process will be presented in the PRISMA flow chart (Fig. 1).

### 2.5. Data extraction

The following data were extracted: lead author; publication year; country of origin; study design; sample size; age; outcome measures and complications. Any differences of opinion will be resolved through group discussion or consultation with a third reviewer. When relevant data is not reported, we will contact the author via email or other means to obtain missing data.

### 2.5. Risk of bias assessment

Two authors will assess methodological quality of included studies separately by the Cochrane collaboration’s risk of bias tool.^[15]^ We will consider the following: random sequence generation (selection bias), allocation concealment (selection bias), blinding of participants and personnel (performance bias), blinding of outcome assessment (detection bias), incomplete outcome data (attrition bias), selective reporting (reporting bias) and other sources of bias (other bias). The bias risk in each aspect will be assessed and divided into 3 levels: low risk, high risk, and unclear risk. The 2 authors will resolve any disagreements through discussion, and will reach consensus through a third reviewer.

### 2.6. Statistical analysis

In this study, we will apply RevMan 5.4 software for statistical analysis. The risk ratio and 95% confidence intervals are collected for enumeration data, while the mean difference or standardized mean difference and 95% confidence intervals are used to calculate continuous outcome data. The heterogeneity of the data is tested by calculating *I*^2^ statistics. The study is not considered to have a large heterogeneity when the *I*^2^ value is <50%. When the *I*^2^ value exceeded 50%, there is significant statistical heterogeneity among the trials. When there is homogeneity in the merged outcome results across sufficient studies, a meta-analysis will be conducted. Otherwise, we performed a subgroup analysis to explore the causes of the heterogeneity.

## 3. Discussion

Osteoporosis is a serious public health concern worldwide because of the morbidity and mortality associated with fragility fracture which is expected to affect a large proportion of people over the age of 50 years. The treatment of osteoporosis consists of lifestyle measures and pharmacologic therapy. Lifestyle measures include adequate vitamin D and calcium, exercise, smoking cessation, counseling on fall prevention, and avoidance of heavy alcohol use.^[16,17]^ These measures should be adopted universally to reduce bone loss. Teriparatide is the first anabolic agent approved for the treatment of patients with osteoporosis and has been reported to reduce the risk of fracture by increasing bone formation.^[18]^ It increases new bone formation by increasing osteoblast differentiation, osteoblast function, and survival.^[19]^ Alendronate is the first bisphosphonate that has been widely used. It inhibits osteoclast-mediated bone resorption, and has been found to increase the BMD.^[20]^ This is the first meta-analysis to evaluate the effectiveness of alendronate and teriparatide in patients with glucocorticoid-induced osteoporosis.

Some limitations should be mentioned. First, the sample size may not large enough to detect the difference between the treatment groups in the incidence of any fracture. Second, since BMD was measured at the lumbar spine, proximal femur, radius, and second metacarpal bone in each institution, the precise analytical methods might have differed among the institutions. Third, there was heterogeneity among studies included in this study, which may affect the results. Additional large-scale randomized controlled trials with a long period of follow-up are required.

## Author contributions

**Data curation:** Shuang Zhang.

**Investigation:** Shuang Wang.

**Writing – original draft:** Na Liang.

**Writing – review & editing:** Juan Ma.
